# Climate change and environmental degradation: Evidence from SADC countries

**DOI:** 10.1371/journal.pone.0346018

**Published:** 2026-04-06

**Authors:** Olanrewaju Adewole Adediran, Samuel Olanrewaju Binuomote, Sotja Graham Dlamini, Daniel Vusanani Dlamini, Joshua Olusegun Ajetomobi, Binganidzo Muchara, Adedolapo Kemi Ajala

**Affiliations:** 1 Department of Sustainable Livelihoods, Graduate School of Business Leadership, University of South Africa, Pretoria, South Africa; 2 Department of Agricultural Economics and Management, University of Eswatini, Luyengo, Eswatini; 3 Department of Agricultural Economics, Ladoke Akintola University of Technology, Ogbomoso, Nigeria; Kumasi Technical University, GHANA

## Abstract

The study investigated the effect of climate change on environmental degradation in the Southern African Development Community (SADC) countries. Using data sourced from the World Bank indicator from 1990 to 2024, the study constructed indices for climate change and environmental degradation using principal component analysis. The study employed Robust Standard Error Estimation in the Pooled Ordinary Least Squares and Fixed-Effects Panel models to examine the study objectives. However, for the sensitivity analysis, the study used a Driscoll-Kraay Standard Error (DKSE) and Panel-Corrected Standard Errors (PCSE) estimators to control for cross-sectional dependence and model heterogeneity. The results revealed that climate change significantly negatively affects the environment and trade openness. A variety of climate hazards critically expose 30% of the SADC region to environmental degradation driven by greenhouse gas emissions, and this may significantly reduce crop productivity without adaptation, especially for cereals. In conclusion, the SADC countries desperately need integrated policies and strategies that promote adaptation and mitigation of climate change, renewable energy, and reduced environmental pollution.

## 1. Introduction

Existing studies indicate that a marginal increase in global temperature will result in significant and unpleasant environmental changes [1]. It is evident that global warming is increasing (+3°C in 2023 according to the World Meteorological Organisation [2] in Africa [3,4]. Global warming has a huge impact on climate change and is adversely affecting agricultural production [5,6]. Climate change refers to ongoing changes in temperatures and weather patterns driven by burning fossil fuels, deforestation, and carbon emissions [7,8]. Environmental degradation, of which greenhouse gas (GHG) emissions are a principal causative factor [9], is the degradation of the natural ecosystem due to the over-depletion of naturally existing resources that support and enhance the lives of both plants and animals. Environmental degradation hampers the survival of various forms of life, threatens their survival, and causes biodiversity loss [10]. Urbanisation, industrialisation and deforestation are major human activities that cause both environmental deterioration and climate change [11]. Despite a large body of research on climate change, there is a paucity of studies that examine climate change and environmental degradation through the lens of the African Continental Free Trade Area (AfCFTA) agreement.

Theoretically, the impact of climate change on environmental degradation is grounded in neoclassical and endogeneity theories. [12] present the environmental Kuznets curve, an inverted U-shaped relationship, applicable to the relationship between climate change and environmental degradation. Intuitively, an increase in economic growth may increase population growth, urbanisation and industrialisation, vice versa. When economic growth (for instance, industrialisation) reaches its peak, it will begin to decline, thereby negatively impacting the environment. In other words, an increase in population, urbanisation, and industrialisation is likely to increase pollution (affecting climate change). In all, the effect of economic growth on climate change and environmental degradation cannot be overemphasised. Notwithstanding, countries have unique growth trajectories that differ over time, even within the same region. Also, policy recommendations to mitigate environmental degradation and climate change have heterogeneous effects in the countries. Variation in policy regulations provides an opportunity to investigate the effect of climate change on environmental degradation through the lens of trade under the AfCFTA agreement.

Empirically, studies have found that climate change has a significant negative effect on human and natural resources [10,13]. It is now almost certain that the southern African social-ecological system, like several other regions of the world, is approaching potentially consequential “tipping points,” sets of conditions that entrain self-perpetuating changes with adverse impacts, predicted to negatively affect SDG-2 [8]. The region has been identified as a global warming hotspot [14] and is expected to be heavily impacted by it [7,15]. Various climate hazards critically expose 30% of the SADC region to land and environmental degradation through greenhouse gas emissions, which, without adaptation, are projected to significantly reduce crop productivity, especially of cereals [16]. The southern African region has experienced a range of climate hazards, which have also significantly impacted agricultural trade and food security [16,17].

The SADC member states, alongside other African countries, became signatories to the African Continental Free Trade Area (AfCFTA) agreement, which was launched in 2018 [18]. One of the objectives of AfCFTA is to influence regional integration and assist trade and investment flows within Africa. It is, however, not yet clear if the AfCFTA implementation in the SADC region has any significant relationship with trade flows, climate change and environmental degradation. While some studies have examined climate change and environmental degradation in Sub-Saharan Africa, there is little or no empirical evidence on the interrelationships between climate change and environmental degradation in the context of implementing the AfCFTA in SADC. Thus, this study provides a pathway to achieving some of the Sustainable Development Goals of the 2030 Agenda in the Southern African region and contributes to both the literature and the global development policy agenda. Therefore, this study's general objective is to examine the effect of climate change on environmental degradation in the South African region. On a specific note, the study evaluated the implications of climate change on environmental degradation and trade. It also examined the effect of climate change and environmental degradation through the lens of AfCFTA (that is, before and after the implementation of AfCFTA).

The remaining part of the study will include Section 2, which explains the data and methods. Section 3 is about econometric strategies. Section 4 consists of the results and their interpretation. Conclusion and recommendations for policy in section 5.

## 2. Data and methods

### 2.1. Data and restructuring of the variables using principal analysis

The study sourced the dataset from the World Bank database (both development indicator – WDI and government indicator – WGI) available at https://data.worldbank.org/. It established a panel data set for Southern African Development Community (SADC) countries. The study covers the period from 1990 to 2024 for analysis. In computing the index of climate change, the study interpolates three variables (such as energy depletion, mineral depletion, and natural resource depletion) with significant missing data points using mipolate. The study standardised the data to avoid spurious results, applied logarithms and first differences, and provided descriptions of other variables (Table 1 summarises the key variables used). Generally, principal component analysis (PCA) is suitable and largely used for restructuring continuous variables. Principal analysis (PCA) is a technique used for data reduction. The steps are as follows: list the variables for the PCA to generate the climate change index from CO_2_ intensity, energy depletion, mineral depletion, natural resources depletion, particulate emission damage, and average precipitation in depth. The study conducted a principal analysis on six variables using principal factor extraction and varimax rotation. The next step in PCA is to rotate the data to obtain factor loadings and to predict the variables. Two factors with eigenvalues greater than 1 were retained, accounting for approximately 63% of the total variance (see S2 Appendix for the output). Hence, the empirical selection of the data identifies two variables, as determined by the component rotation matrix (with higher loadings from component 1: CO2 intensity and particulate emission damage), as drivers of climate change. Communalities indicate that the retained factors adequately represent all variables. The Kaiser-Meyer-Olkin (KMO) threshold value is 50%; the KMO value for the climate change index is 50%.

**Table 1:** Variable Description and Source.

Variables	Description	Indicator	Source	Standardisation
Climate Change	Index Generated through Factor Analysis	Independent variable	WDI, 2024	Logarithm
Environmental degradation	Index Generated through Principal Component Analysis	Dependent variable	WDI, 2024	Logarithm
Trade	Trade (% of GDP)	Dependent variable	WDI, 2024	Logarithm
CO2 Emission	Carbon dioxide (CO2) emissions excluding LULUCF per capita (t CO2e/capita)	Independent variable	WDI, 2024	Logarithm
Industrialisation	Manufacturing, value added (% of GDP)	Control Variable (CV)	WDI, 2024	Logarithm
Urbanisation	Population in the largest city (% of urban population)	CV	WDI, 2024	Logarithm
Inflation rate	Inflation, GDP deflator (annual %)	CV	WDI, 2024	Logarithm
Exchange-rate	Real effective exchange rate index	CV	WDI, 2024	Logarithm
GDP growth rate	GDP per capita growth (annual %)	CV	WDI, 2024	Logarithm
Renewable energy consumption	(% of total final energy consumption)	CV	WDI, 2024	Logarithm
Foreign direct investment	Net inflows (% of GDP)	CV	WDI, 2024	Logarithm
Government regulations	Index Generated through Principal Component Analysis	CV	WGI, 2024	Logarithm

Authors’ compilation, 2026; For the climate change and environmental degradation index, the created variables are standardised before use.

A similar process was repeated for the environmental degradation indices for agricultural land, arable land, forest area, net forest depletion, annual freshwater withdrawal, and CO2 emissions, using principal component analysis. Following the Kaiser rule, only factors with eigenvalues greater than 1 should be retained. The first two components, with eigenvalues greater than 1, were retained, accounting for 83% of the total variance (see S3 Appendix for the output). Empirically, the study selected agricultural land and CO2 emissions to generate the index of environmental degradation. The Kaiser-Meyer-Olkin (KMO) value for the environmental degradation index is 50%. Government regulatory quality or institutional factors consist of variables such as control of corruption, government effectiveness, regulatory quality, rule of law, political stability, and voice and accountability. All missing data points were populated using mipolate.

### 2.2. Unit root test

The study conducted a unit root test to assess the dataset's stationarity. Table 2 presents unit root tests for the variables using the [19] unit root test and the Fisher-type tests of [20]. Climate change, environmental degradation, and trade indices were used as level indices, while other variables were used as first differences.

**Table 2:** Unit root test.

Variables	Im–Pesaran–Shin unit-root		Fisher-type unit-root test	
	Level	1^st^ difference	Level	1^st^ difference
Log GDPpcg	−6.3099***	−13.6333***	−9.9587***	−42.4912***
Log Trade	−1.3683	−12.1111	−1.8007***	−31.7765***
Log Urbanisation	2.4198	−5.9515***	1.3816	−7.3807***
Log Inflation rate	−6.3437***	−14.067***	−51.815***	−51.815***
Log Exchange rate	−3.1886***	−9.9465***	−7.2084***	−19.8325***
Population	−3.0907***	−8.859***	−7.9006***	−23.2183***
Log FDI	−6.6963***	−15.0544***	−11.1877***	−53.5722***
Log Industrialisation	0.0108	−13.2523	0.4395***	−32.2278***
Log Renewable energy	2.7911	−11.8173	1.3534***	−29.2589***

*, **, *** implies series stationary significant level at 10%, 5%, 1% respectively; Author’s Computation 2026.

### 2.3. Test of normality

**Fig 1:**
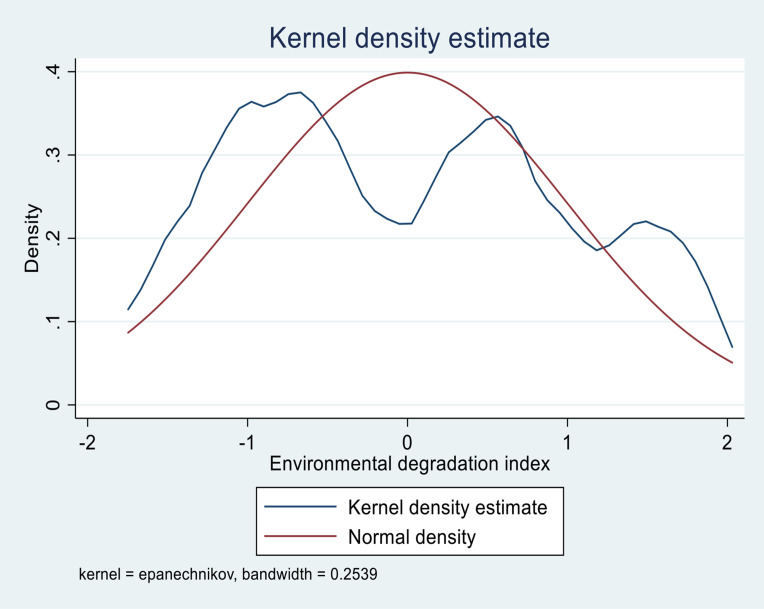
Kernel density of Environmental degradation, Source: Computed by the Authors’ 2026.

**Fig 2:**
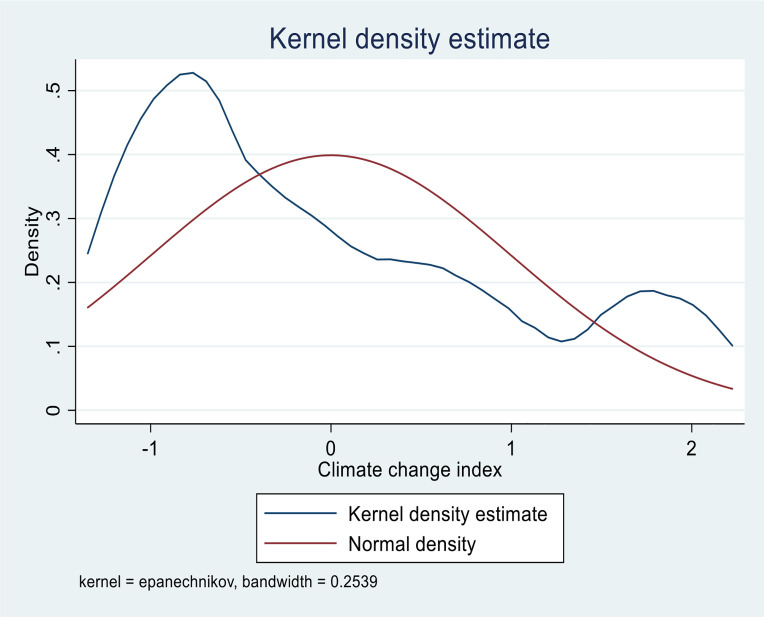
Kernel density of Climate change, Source: Computed by the Authors’ 2026 The study presents the summary statistics in the next paragraph.

Furthermore, we perform normality tests using kernel density analysis for key variables. Fig 1 denotes the test of normality using the Epanechnikov kernel density estimates of the environmental degradation index. The environmental degradation index kernel density is symmetrical and left-skewed. It is not smooth along with the bell curve, suggesting that the dataset may be normally distributed. Stationarity may not be required for the index created. Also, the kernel density in Fig 2 shows that the climate change index computed by PCA is more evenly distributed. The data distribution follows a bell curve, indicating that the dataset is symmetrical and that the variable is normally distributed.

### 2.4. Summary statistics

Table 3 presents descriptive statistics of the sustainable development index, climate change index, and other variables for the panel countries. In the panel dataset, the environmental degradation mean is zero (0), and the standard deviation (SD) is one (1). The environmental degradation ranges between −1.49 and 1.78. Also, for the constructed climate change index, the mean is near zero (0), and the standard deviation is near one (1). The climate change index ranges from −1.09 to 1.97. All variables range from 0.1 to 1 digit. The minimum and maximum revealed that there is no obvious outlier in the variables.

**Table 3:** Descriptive Statistics.

Variable	Obs	Mean	Std. Dev.	Min	Max
Environmental Degradation	560	0	1	−1.493	1.777
Climate Change	560	0	1	−1.094	1.971
GDP per capita growth	560	0.905	0.336	0.126	1.555
Trade openness	560	4.428	0.072	4.31	4.576
Urbanisation	560	3.397	0.006	3.387	3.409
Exchange rate	560	5.445	0.509	4.43	6.215
Foreign Direct Investment	560	1.17	0.454	0.211	2.154
Inflation rate	560	3.306	1.416	1.511	7.511
Industrialisation	560	2.573	0.14	2.402	2.846
Renewable energy	560	4.088	0.1016	3.707	4.42
Population	560	2.069	0.231	1.695	2.765
Corruption	560	3.587	0.2	2.616	3.757
Government	560	3.801	0.157	2.991	3.909
Political Stability	560	3.591	0.105	3.055	3.701
Regulatory Quality	560	3.64	0.132	2.937	3.727
Rule of Law	560	3.704	0.03	3.631	3.752

*Note: Log variables: (*Trade openness, Urbanisation, Exchange rate, Manufacturing, GDP per capita growth, Renewable energy, Inflation rate, Foreign Direct Investment, Corruption, Government, Political Stability, Regulatory Quality, Rule of Law, Voice Accountability*); Authors’ computation, 2026.*

## 3. Econometric strategies

In econometric analysis, especially in panel datasets, diagnostic tests are very important and must be met to ensure the validity and reliability of the model estimates. Diagnostic tests are essential for addressing econometric issues such as multicollinearity, cross-sectional dependence, heteroscedasticity and autocorrelation. These tests help to enhance the credibility of the findings and ensure that the estimated results reflect the true relationships between the variables.

This study employed the robust standard-error fixed-effects estimator, eliminating time-invariant unobserved errors specific to each observation. For a robustness check, we employ a regression with Driscoll-Kraay standard errors estimator [21]. The methodologies have provisions for correcting potential serial correlation and heteroscedasticity across panels, and using appropriate standard errors is crucial to making accurate statistical inferences [22]. Following [22], the overall functional model is specified in equation (1) as follows:


$$Y_{it}=\beta_{\text{0}}+\beta_{\text{1}}X_{it}+\beta_{\text{2}}Z_{it}+\varepsilon_{it}$$ (1)

Where; $Y_{it}$ is a vector of dependent variables, such as environmental degradation (EDG) and trade ${TRAD}_{it}$ indices at time t and country $i.{\;X}_{it}$ represents the major independent variables, such as the climate change index (CCH) at the time, t and country, i. $Z_{it}$ represents a vector of other important control variables, such as industrialisation (IND), the inflation rate (INFL), the exchange rate (EXR), renewable energy (RENE), foreign direct investment (FDI), and gross domestic product per capita growth (GDPpcg). $\varepsilon_{it}$ is the error term.


$${\text{EDG}}_{it}=\beta_{\text{0}}+\beta_{\text{1}}{CCH}_{it}+\beta_{\text{2}}{\text{GDPpcg}}_{it}+\beta_{\text{3}}{\text{IND}}_{it}+\beta_{\text{4}}{\text{RENE}}_{it}+\beta_{\text{5}}{FDI}_{it}+\beta_{\text{6}}{POP}_{it}+\varepsilon_{it}$$ (2)

Also, to evaluate the implications of climate change and environmental degradation for the success of the AfCFTA, we examined their effects on trade openness in SADC by treating trade as the dependent variable, following the established literature on trade and environment (e.g., [23]). The linear model is therefore specified as:


$${TRAD}_{it}=\beta_{\text{0}}+\beta_{\text{1}}{CCH}_{it}+\beta_{\text{2}}{\text{RENE}}_{it}+\beta_{\text{3}}{\text{IND}}_{it}+\beta_{\text{4}}{INFL}_{it}+\beta_{\text{5}}{EXR}_{it}+\beta_{\text{6}}{FDI}_{it}+\beta_{\text{7}}{\text{GDPpcg}}_{it}+\varepsilon_{it}$$ (3)

## 4. Empirical results

### 4.1. Correlation and variance inflation factor tests for multicollinearity

A correlation matrix is a statistical tool that shows how strongly and in what direction two or more variables are related. The matrix shows how all the possible pairs of values in a table are related to each other. It is a powerful tool for summarising a large dataset and for finding and showing patterns in the data.

Table 4 presents a correlation analysis of environmental degradation, the climate change index, and other control variables. The correlation analysis indicates that the Environmental degradation and climate change index (r = −0.74) may be negatively correlated. Also, environmental degradation and GDP per capita growth may be negatively correlated (r = −0.11). The environmental degradation index and renewable energy are negatively correlated (r = −0.43). The correlation between the environmental degradation index and industrialisation may be negatively related (r = −0.64). The environmental degradation index and population may be negatively related (r = −0.29). The correlation analysis indicates that environmental degradation and FDI may be positively correlated (r = 0.57). The rule of the Variance Inflation Factor (VIF) is that the values must not exceed 10. Otherwise, it indicates high multicollinearity in the dataset, which can lead to instability in the regression coefficients or higher standard errors. Table 4 above presents the VIF Analysis to test for multicollinearity among environmental degradation, climate change, and other control variables. The results show that there is no indication of multicollinearity between environmental degradation, climate change index and other variables.

**Table 4:** Matrix of correlations and Variance inflation factor.

Variables	VIF	1/VIF	(1)	(2)	(3)	(4)	(5)	(6)	(7)
(1) Environmental degradation			1.000						
(2) Climate change	7.456	0.134	−0.744	1.000					
(3) GDP per capita growth	1.162	0.86	−0.110	0.232	1.000				
(4) Renewable energy	1.146	0.873	−0.432	0.311	0.121	1.000			
(5) Foreign direct investment	1.928	0.519	0.573	−0.690	−0.175	−0.149	1.000		
(6) Industrialisation	4.654	0.215	−0.643	0.857	0.322	0.284	−0.537	1.000	
(7) Population	2.123	0.471	−0.291	0.596	−0.046	0.059	−0.523	0.348	1.000
Mean VIF	1.678								

Authors’ computation, 2026.

### 4.2. Cointegration estimations

Cointegration statistics is an analysis that estimates the long-run relationship between two variables [24–27]. In other words, it examines the long-run relationship between variables in the panel dataset. The study presents a cointegration test comprising Kao's residual cointegration test [24], Pedroni's residual cointegration test [26], and Westerlund’s cointegration test [27] (see Table 5). The study performs a cointegration estimation to determine whether the variables are cointegrated. Using a linear combination of the sustainable development index and climate change. We use multiple cointegration tests to assess the consistency of the results. The results indicate cointegration, suggesting a long-run relationship between climate change and environmental degradation.

**Table 5:** Cointegration analysis.

	Environmental degradation model			Trade openness model		
	KAO Stat.	Pedroni Stat.	Westerlund Stat.	KAO Stat.	Pedroni Stat.	Westerlund Stat.
Modified Dickey-Fuller t	−8.955***			−36.103***		
Dickey-Fuller t	−5.430***			−20.360***		
Augmented Dickey-Fuller t	−7.771***	−0.639		−16.272***	−7.794***	
Unadjusted modified Dickey Fuller t	−8.955***			−36.103***		
Unadjusted Dickey-Fuller t	−5.430***			−20.360***		
Modified Phillips-Perron t		2.691***			0.912	
Phillips-Perron t		−0.919			−7.598***	
Variance ratio		−3.035***	2.752***		−1.707**	−1.545*

*, **, *** implies series stationary significant level at 10%, 5%, 1% respectively; Authors’ computation, 2026.

### 4.3. Cross-sectional dependence test

One of the main problems in using panel data is cross-sectional dependence (CSD). According to [28], a cross-sectional dependence test was generated to test for cross-sectional dependence in large (N) and small (T) panels. Breusch-pagan Lagrange multiplier, Pesaran scaled Lagrange multiplier, and Pesaran cross-sectional dependence (CD) test were used. In Table 6, the study conducts a cross-sectional dependence test (CSD Test) by [28], which indicates no cross-sectional dependence for all variables for both models.

**Table 6:** Cross-sectional dependence.

	Environmental degradation model			Trade openness model		
Variables	alpha	Std. Err.	[95% Conf. Interval]	alpha	Std. Err.	[95% Conf. Interval]
Trade openness				1.0052	0.0389	0.9290 - 1.0814
Environmental degradation	1.0052	0.0549	0.8977 - 1.1128			
Climate change	1.0052	0.0619	0.8839 - 1.12658	1.0052	0.0619	0.8839 - 1.1266
GDP pc growth	1.0052	0.0272	0.9519 - 1.0586	1.0052	0.0272	0.9519 - 1.0586
Renewable energy	1.0052	0.3269	0.3646 - 1.6459	1.0052	0.3269	0.3646 - 1.6459
FDI	1.0052	0.0466	0.9139 - 1.0965	1.0052	0.0466	0.9139 - 1.0965
Industrialisation	1.0054	0.0433	0.9206 - 1.0902	1.0054	0.0433	0.9206 - 1.0902
Population	1.0052	0.0462	0.9148 - 1.0957			
Exchange rate				1.0052	0.5933	−0.1577 - 2.1681
Inflation rate				1.0052	0.0634	0.881 - 1.1295

0.5 <*=* alpha < 1 implies strong cross-sectional dependence; Variables are centered around zero.; Authors’ computation, 2026.

### 4.4. Pesaran and Yamagata (2008) slope homogeneity test

Further, the study performed a slope homogeneity test following [29] in a panel regression mode. It may be assumed that the slope of coefficients in the panel model is homogeneous across individuals [30,31]. However, heteroskedasticity bias may arise when the homogeneity assumption is violated, leading to panel estimates with heterogeneous slopes [32].

Table 7 shows the results of the [29] test for slope heterogeneity. This implies that the slope coefficients differ across the cross-sectional panels in the model. The study employed a panel-corrected standard error estimator to account for cross-sectional dependence and heterogeneity.

**Table 7:** Pesaran and Yamagata (2008) Slope Homogeneity Test.

Test	Environmental Degradation Statistic	Trade Openness Statistic
∆Test	−7.483***	−8.000***
∆ Adj. Test	−8.727***	−9.522***

Standard errors in parentheses: *** p < 0.01, ** p < 0.05, * p < 0.1; Authors’ computation, 2026.

### 4.5. Climate change on environmental degradation: Robust fixed effect estimators

The study decided not to include urbanisation in the model because its inclusion produces questionable results, and we are unable to explain them (see S1 Appendix for the result output with urbanisation, but without the time effect).

Table 8 presents robust standard errors from the fixed-effects estimator for cases with and without the African Continental Free Trade Agreement regime. Column (1) shows that climate change has a significant negative impact on environmental degradation using the fixed-effect (FE) estimator. The current study's results are consistent with those of [10], who found that climate change contributes to environmental degradation. Climate change is likely to have adverse effects on the environment, leading to degradation, which implies that a low- or lower-quality climate would negatively impact the environment. The study included some covariates in the Columns (2) estimation. Column (2) revealed that climate change had a significant negative impact on environmental degradation, as indicated by FE. The foreign direct investment (FDI) has a significant negative effect on environmental degradation. The population has a significant positive impact on environmental degradation. Also, economic growth (GDP per capita growth) has a significant positive effect on environmental degradation.

**Table 8:** Impact of Climate Change on Environmental Degradation: POLS and Fixed Effect Estimator.

	(1)	(2)	(3)	(4)	(5)
	Fixed effect	Fixed effect	Pool OLS robust	Pool OLS robust	Pool OLS robust
	With one variable	Panel	Baseline Panel	Before AfCFTA	After AfCFTA
Variables	Environmental Degradation	Environmental Degradation	Environmental Degradation	Environmental Degradation	Environmental Degradation
Climate change	−0.221***	−1.397***	−3.299***	−1.142***	−1.536***
	(1.47e-09)	(3.47e-09)	(3.93e-10)	(9.23e-07)	(6.35e-11)
GDP pc growth		0.745***	0.912***	0.522***	−0.157***
		(2.91e-08)	(2.83e-10)	(3.72e-07)	(0)
FDI		−1.354***	−1.801***	−1.207***	−0.167***
		(1.02e-07)	(6.32e-11)	(4.69e-07)	(0)
Population		0.449***	0.922***	1.475***	1.210***
		(1.32e-07)	(5.00e-10)	(3.74e-08)	(5.73e-11)
Renewable energy			13.97***	−0.643***	11.04***
			(7.76e-09)	(6.06e-07)	(1.45e-09)
Industralisation			6.149***	−0.824***	−1.382***
			(7.67e-10)	(5.30e-06)	(1.82e-10)
Year dummy	Yes	Yes	Yes		
Y_1990–2017				Yes	
Y_2018–2024					Yes
Constant	−1.054***	−0.430***	−73.52***	1.816***	−43.42***
	(4.58e-09)	(3.97e-07)	(3.04e-08)	(1.60e-05)	(5.52e-09)
Observations	560	560	560	560	560
R-squared	1.000	0.923		0.818	
Number of id	16	16	16	16	16
sigma_u	0	0	0	0	0
sigma_e	0.00302	0.289	0.235	0.444	0.349
rho	0	0	0	0	0

Robust standard errors in parentheses: *** p < 0.01, ** p < 0.05, * p < 0.1; FDI implies Foreign direct investment; Authors’ computation, 2026.

Column (3) presents that climate change has a significant negative impact on environmental degradation. Foreign direct investment (FDI) has a significant negative impact on environmental degradation. A proportional increase in population may influence environmental degradation. A significant positive increase in industrialisation is likely to increase economic growth, and environmental degradation may also increase. Columns (4) and (5) presented estimates of the impact of climate change on environmental degradation before and after the African Continental Free Trade Area (AfCFTA), respectively.

In Column (4), before the implementation of AfCFTA, climate change had a significant negative impact on environmental degradation. Economic growth (GDP per capita growth) has a significant positive effect on environmental degradation. Foreign direct investment (FDI) has a significant negative impact on environmental degradation. The population has a significant positive effect on environmental degradation. Renewable energy has a significant negative impact on environmental degradation. Industrialisation has a significant negative impact on environmental degradation. This is consistent with the findings of a recent study by [33], which reveals that industrial waste can contribute to environmental degradation.

After the implementation of AfCFTA in Column (5), climate change had a significant negative impact on environmental degradation. Economic growth (GDP per capita growth) has a significant adverse effect on environmental degradation. Foreign direct investment (FDI) has a significant negative impact on environmental degradation. The population has a significant positive impact on environmental degradation. Renewable energy has a significant positive effect on degradation. Industrialisation has a significant negative impact on environmental degradation.

Table 9 Column (1) presents that climate change significantly negatively impacts trade openness with the FE estimate. In Column (2), climate change has a significant negative impact on environmental degradation, as indicated by FE. Also, economic growth (GDP per capita growth) has a significant positive effect on trade openness. Industrialisation has a significant positive impact on environmental degradation. At the same time, foreign direct investment (FDI) has a significant adverse effect on trade openness.

**Table 9:** Impact of Climate Change on Trade: Fixed effect estimator.

	(1)	(2)	(3)	(4)	(5)
	Fixed effect	Fixed effect	Pool OLS robust	Pool OLS robust	Pool OLS robust
	With one variable	Panel	Baseline Panel	Before AfCFTA	After AfCFTA
Variables	Trade openness	Trade openness	Trade openness	Trade openness	Trade openness
Climate change	−0.0271***	−0.141***	−0.0270***	−0.130***	−0.149***
	(3.62e-10)	(2.04e-06)	(2.59e-10)	(9.59e-11)	(0)
GDP pc growth		0.0200***	0.00982***	0.00187***	0.0356***
		(5.26e-07)	(1.32e-10)	(0)	(0)
FDI		0.00344***	0.0445***	0.0972***	0.0697***
		(4.59e-07)	(1.69e-10)	(0)	(0)
Industralisation		0.837***	−0.891***	0.741***	0.0399***
		(1.46e-05)	(0)	(7.66e-10)	(0)
Exchange rate			−0.255***	−0.123***	−0.183***
			(4.87e-10)	(0)	(0)
Inflation rate			−0.0274***	−0.0426***	0.0149***
			(0)	(0)	(0)
Renewable energy			−1.483***	−0.288***	0.292***
			(2.28e-09)	(1.49e-10)	(1.29e-10)
Year dummy	Yes	Yes	Yes		
Y_1990–2017				Yes	
Y_2018–2024					Yes
Constant	4.415***	2.252***	14.31***	4.380***	3.993***
	(1.00e-08)	(3.74e-05)	(1.20e-08)	(2.74e-09)	(4.32e-10)
Observations	560	560	560	560	560
R-squared	0.999	1.000			
Number of id	16	16	16	16	16
sigma_u	0	0	0	0	0
sigma_e	0.00232	0.000373	0.00537	0.00122	0.0257
rho	0	0	0	0	0

Robust standard errors in parentheses: *** p < 0.01, ** p < 0.05, * p < 0.1; Authors’ computation, 2026.

Column (3) shows that climate change has a significant negative impact on trade openness. Economic growth has a significant positive impact on trade openness, consistent with previous studies [34,35], which show that endogenous growth models are positively associated with trade openness. Foreign direct investment (FDI) has a significant negative impact on trade openness [36]. In contrast, industrialisation has a significant negative impact on trade openness. The inflation rate has a significant negative impact on trade [33], and the exchange rate also has a significant negative impact [37]. Renewable energy has a significant negative impact on trade openness.

The study further examines the effect of climate change on trade through the lens of the AfCFTA agreement. In Column (4), before the implementation of the AfCFTA agreement, climate change significantly reduced trade openness. Economic growth has a significant positive impact on trade openness, and foreign direct investment (FDI) has a significant positive impact on trade. Industrialisation has a significant positive impact on trade. At the same time, the exchange rate has a significant negative impact. Also, renewable energy and the inflation rate have a significant negative impact on trade.

In Column (5), after the implementation of the AfCFTA agreement, climate change significantly negatively impacts trade openness. Economic growth and FDI are likely to have a significant positive effect on trade openness. Industrialisation and Renewable energy have a significant positive impact on trade. The inflation rate has a significant positive impact on trade. In contrast, the exchange rate has a significant negative impact.

### 4.6. Impact of climate change on environmental degradation with governance qualities

Furthermore, the study investigates the effect of institutional factors in the estimation of the impact of climate change on environmental degradation and trade openness, using a robust panel ordinary least squares (POLS) estimator.

Table 10 column (1) shows that climate change has a significant negative impact on environmental degradation, with corruption included. GDP per capita growth may have a significant positive impact on environmental degradation. Also, foreign direct investment and renewable energy significantly affect environmental degradation. At the same time, industrialisation and corruption have a significant positive effect on environmental degradation.

**Table 10:** Environmental degradation and Climate change with Governance Quality.

	(1)	(2)	(3)	(4)	(5)	(6)
Variables	Environmental degradation	Environmental degradation	Environmental degradation	Environmental degradation	Environmental degradation	Environmental degradation
Climate change	−2.890***	−4.004***	−3.859***	−3.141***	−4.140***	−0.232***
	(0)	(2.14e-10)	(3.23e-10)	(5.14e-11)	(2.71e-10)	(9.00e-10)
GDP pc growth	1.488***	1.084***	0.961***	1.281***	1.361***	0.861***
	(0)	(0)	(1.01e-10)	(0)	(0)	(2.99e-10)
Renewable energy	−0.267***	6.469***	8.325***	3.398***	9.324***	−8.969***
	(4.93e-10)	(9.62e-10)	(2.31e-09)	(1.23e-10)	(1.78e-09)	(2.27e-09)
FDI	−1.981***	−2.883***	−2.220***	−2.777***	−2.553***	−2.615***
	(0)	(1.26e-10)	(8.62e-11)	(0)	(1.17e-10)	(1.72e-10)
Industrialisation	8.781***	9.197***	9.264***	6.917***	10.24***	−3.105***
	(9.21e-11)	(4.80e-10)	(4.95e-10)	(2.23e-10)	(5.05e-10)	(4.52e-09)
Corruption	1.350***					
	(2.94e-10)					
Government		4.630***				
		(4.68e-10)				
Political Stability			6.669***			
			(1.39e-09)			
Regulatory Quality				5.134***		
				(7.69e-11)		
Rule of Law					8.157***	
					(1.17e-09)	
Voice Accountability						−43.25***
						(1.44e-08)
Year dummy	Yes	Yes	Yes	Yes	Yes	Yes
Constant	−25.91***	−64.61***	−81.51***	−48.47***	−92.38***	206.2***
	(2.84e-09)	(6.67e-09)	(1.57e-08)	(1.33e-09)	(1.26e-08)	(7.44e-08)
Observations	560	560	560	560	560	560
Number of id	16	16	16	16	16	16
sigma_u	0	0	0	0	0	0
sigma_e	0.263	0.234	0.237	0.235	0.230	0.0918
rho	0	0	0	0	0	0

Robust standard errors in parentheses: *** p < 0.01, ** p < 0.05, * p < 0.1; Authors’ computation, 2026.

Column (2) shows that climate change has a significant negative impact on environmental degradation, with the inclusion of governance. GDP per capita growth, industrialisation, and renewable energy have a significant positive impact on environmental degradation. Governance has a significant positive effect on environmental degradation.

Column (3) shows that climate change has a significant negative impact on environmental degradation even when political stability is included. GDP per capita growth, industrialisation, and renewable energy had a significant positive impact on environmental degradation. FDI has a significant negative effect on environmental degradation.

Column (4) shows that climate change has a significant negative impact on environmental degradation, even when regulation quality is included. FDI has a significant negative impact on environmental degradation. GDP per capita growth, renewable energy and industrialisation have a significant positive impact on environmental degradation.

Column (5) shows that climate change has a significant negative impact on environmental degradation, even when the rule of law is taken into account. GDP per capita growth, industrialisation, and renewable energy have a significant positive impact on environmental degradation. The FDI has a significant negative effect on environmental degradation.

Column (6) shows that climate change has a significant negative impact on environmental degradation, even when voice accountability is taken into account. GDP per capita growth has a significant positive impact on environmental degradation. FDI, industrialisation, renewable energy, and voice accountability have a significant adverse effect on environmental degradation.

The study further includes institutional quality or factors in the analysis of the effect of climate change on trade openness. Table 11 column (1) to (6) shows that climate change has a significant negative impact on trade openness. Similarly, FDI surprisingly negatively affected trade openness (see columns 1–6 in Table 11). Notwithstanding, GDP per capita growth and industrialisation may have a significant positive impact on trade openness. All institutional quality indicators (such as control of corruption, governance, political stability, regulatory quality, rule of law, and voice and accountability) negatively affect trade openness to varying degrees.

**Table 11:** Trade Openness and Climate Change with Governance Quality.

	(1)	(2)	(3)	(4)	(5)	(6)
Variables	Trade	Trade	Trade	Trade	Trade	Trade
Climate change	−0.153***	−0.154***	−0.154***	−0.156***	−0.154***	−0.152***
	(0)	(0)	(0)	(0)	(0)	(0)
GDP pc growth	0.0311***	0.0305***	0.0315***	0.0290***	0.0312***	0.0383***
	(0)	(0)	(0)	(0)	(0)	(0)
FDI	−0.00405***	−0.00400***	−0.00372***	−0.00277***	−0.00428***	−0.00582***
	(0)	(0)	(0)	(0)	(0)	(0)
Industrialisation	0.923***	0.934***	0.926***	0.949***	0.929***	0.890***
	(8.20e-11)	(7.27e-11)	(8.44e-11)	(9.81e-11)	(7.71e-11)	(0)
Corruption	−0.0117***					
	(0)					
Government		−0.0102***				
		(0)				
Political Stability			−0.0119***			
			(0)			
Regulatory Quality				−0.0186***		
				(0)		
Rule of Law					−0.0134***	
					(0)	
Voice Accountability						−0.0695***
						(0)
Year dummy	Yes	Yes	Yes	Yes	Yes	Yes
Constant	2.072***	2.036***	2.065***	2.028***	2.062***	2.368***
	(1.83e-10)	(1.63e-10)	(1.84e-10)	(1.98e-10)	(1.66e-10)	(1.69e-10)
Observations	560	560	560	560	560	560
Number of id	16	16	16	16	16	16
sigma_u	0	0	0	0	0	0
sigma_e	0.00104	0.00109	0.00107	0.00108	0.00105	0.00114
rho	0	0	0	0	0	0

Robust standard errors in parentheses: *** p < 0.01, ** p < 0.05, * p < 0.1; Industrialisation denote Manufacturing value added; Authors’ computation, 2026.

### 4.7. Sensitivity analysis: Regression with Driscoll-Kraay standard errors and panel-corrected standard errors

The study proposed using Driscoll–Kraay Standard Errors (DKSE) and Panel-Corrected Standard Errors (PCSE) to correct for heteroskedasticity and cross-sectional dependence arising from regional spillovers. The PCSE model also addresses serial autocorrelation by applying the Prais-Winsten regression to correct for first-order serial correlation in panel data residuals [38,39]. The PCSE is designed for panels with a moderately long time (less than 15–20 years). Driscoll–Kraay Standard Errors (DKSE) approach was designed to correct econometric issues such as heteroskedasticity, cross-sectional dependence and autocorrelation [40,41]. In addition, DKSE is suitable for a panel analysis when the time is greater than 20 years (over 30). Hence, both PCSE and DKSE are included in the current study to enhance robustness.

Table 12 presents the analysis of the effect of climate change on environmental degradation by applying PCSE and DKSE estimators.

**Table 12:** Environmental degradation and climate change: DKSE and PCSE.

	(1)	(2)	(3)	(4)	(5)	(6)
	Driscoll–Kraay Standard Errors (DKSE			Panel-Corrected Standard Errors (PCSE)		
	Baseline Panel	Before AfCFTA	After AfCFTA	Baseline Panel	Before AfCFTA	After AfCFTA
Variables	Environmental degradation	Environmental degradation	Environmental degradation	Environmental degradation	Environmental degradation	Environmental degradation
Climate change	−1.144***	−1.369**	−1.193***	−1.133***	−1.166***	−1.202***
	(0.409)	(0.608)	(0.0673)	(0.184)	(0.225)	(0.0474)
GDP pc growth	1.336	0.361	−0.175***	1.347***	0.466	−0.173**
	(0.966)	(0.903)	(0.0640)	(0.434)	(0.445)	(0.0852)
FDI	−1.173	−1.822*	−0.241**	−1.142*	−0.927*	−0.257***
	(0.873)	(0.943)	(0.0970)	(0.635)	(0.546)	(0.0923)
Population	0.0983	1.284	0.660***	0.0997	1.253	0.672***
	(1.116)	(1.530)	(0.124)	(0.661)	(0.842)	(0.148)
Year dummy	Yes			Yes		
Y_1990–2017		Yes			Yes	
Y_2018–2024			Yes			Yes
Constant	−0.647	−1.804	−0.652*	−0.694	−2.561	−0.660*
	(2.250)	(3.699)	(0.364)	(1.837)	(2.067)	(0.382)
Observations	560	560	560	560	560	560
R-squared				0.925	0.689	0.978
Number of groups	16	16	16			
Number of id				16	16	16

Standard errors in parentheses: *** p < 0.01, ** p < 0.05, * p < 0.1; Authors’ computation, 2026.

Table 12 Column (1) presents that climate change has a significant negative impact on environmental degradation using the DKSE technique. Before the implementation of AfCFTA, climate change had a significant negative impact on environmental degradation in Table 11 Column (2). Foreign direct investment (FDI) has a significant negative impact on environmental degradation. After the implementation of AfCFTA in Column (3), climate change had a significant negative impact on environmental degradation. Economic growth (GDP per capita growth) has a significant negative effect on environmental degradation. Foreign direct investment (FDI) has a significant negative impact on environmental degradation. The population has a significant positive impact on environmental degradation.

Column (4) shows that climate change has a significant negative impact on environmental degradation by employing the PCSE estimator. Economic growth (GDP per capita growth) has a significant negative effect on environmental degradation. Foreign direct investment (FDI) has a significant negative impact on environmental degradation. The population has a significant positive effect on environmental degradation. Before the AfCFTA regime, climate change had a significant negative impact on environmental degradation, as shown in Column (5). Also, foreign direct investment (FDI) has a significant negative impact on environmental degradation. In Column (6), during the AfCFTA period, climate change, FDI, and GDP had a significant negative impact on environmental degradation. Whereas the population has a significant positive effect on environmental degradation.

Table 13 presents the impact of climate change on trade openness, using the Driscoll–Kraay Standard Errors (DKSE) estimator and impact before and after the implementation of the AfCFTA agreement.

**Table 13:** Trade openness and climate change: DKSE and PCSE.

	(1)	(2)	(3)	(4)	(5)	(6)
	Driscoll–Kraay Standard Errors (DKSE)			Panel-Corrected Standard Errors (PCSE)		
	Baseline Panel	Before AfCFTA	After AfCFTA	Baseline Panel	Before AfCFTA	After AfCFTA
Variables	Trade	Trade	Trade	Trade	Trade	Trade
Climate change	−0.00638	−0.0421	−0.0427***	−0.00809***	−0.0466	−0.0421***
	(0.00624)	(0.0575)	(0.0139)	(0.00314)	(0.0284)	(0.0115)
GDP pc growth	0.0230***	0.0212	0.0651***	0.0161*	0.0160	0.0659***
	(0.00822)	(0.0550)	(0.0153)	(0.00878)	(0.0303)	(0.0153)
FDI	0.204***	0.126**	0.0797***	0.195***	0.111***	0.0811***
	(0.0236)	(0.0462)	(0.0207)	(0.0105)	(0.0350)	(0.0163)
Industrialisation	0.421***	0.453	0.0876	0.423***	0.458***	0.0864
	(0.0424)	(0.352)	(0.0553)	(0.0191)	(0.178)	(0.0683)
Year dummy	Yes			Yes		
Y_1990–2017		Yes			Yes	
Y_2018–2024			Yes			Yes
Year dummy	Yes			Yes		
Constant	3.091***	3.108***	4.059***	3.100***	3.116***	4.059***
	(0.109)	(0.893)	(0.142)	(0.0485)	(0.455)	(0.173)
Observations	560	560	560	560	560	560
R-squared				1.000	0.969	0.875
Number of groups	16	16	16			
Number of id				16	16	16

Standard errors in parentheses: *** p < 0.01, ** p < 0.05, * p < 0.1; Authors’ computation, 2026.

Table 13 Column (1) presents that GDP, FDI and industrialisation have a significant negative impact on trade openness using the DKSE technique. In column (2), foreign direct investment (FDI) has a significant negative impact on trade openness, before entering the AfCFTA agreement. In Column (3), climate change had a significant negative impact on environmental degradation after the implementation of the AfCFTA agreement. Economic growth (GDP per capita growth) and Foreign direct investment (FDI) have a significant negative effect on trade openness. The results of applying the PCSE estimator to investigate the impact of climate change on trade openness are consistent with those of the DKSE estimator. The results are similar except for column (5), where industrialisation influences trade openness. This is consistent with previous studies, which found that trade and industrialisation are positively correlated [42,43].

### 4.8. Discussion

The study examines the impact of climate change on environmental degradation in South Africa, an area that is scarcely researched. The findings revealed that climate change has a significant negative impact on environmental degradation. Previous studies have indicated that climate change is likely to affect biodiversity by intensifying severe weather and worsening environmental degradation [44,45]. Economic growth (GDP per capita growth) has a significant positive effect on environmental degradation. This implies that the increase in GDP may have a causal effect on environmental degradation, which is consistent with previous findings [46–48]. The environmental Kuznets curve (inverted-U shape) indicates that as gross domestic product per capita increases, the environmental degradation changes [47,49,50].

The foreign direct investment (FDI) has a significant negative effect on environmental degradation. This might not have happened without functional institutional policy [51] and utilising the inflow of FDI to strike a balance between economic growth and the environment [52]. An increase in FDI inflows may accelerate industrialisation, enhance economic growth, but negatively impact environmental degradation. The current finding is inconsistent with the study by [53] in Romania, which found a linear positive relationship between FDI and environmental indicators. Another study found that FDI has an insignificant positive impact on environmental degradation in South Africa, based on a panel study of selected countries [54]. The inflow of FDI may increase if there is political stability, infrastructure and access to resources [53]. There is an inconsistency in political stability across SADC countries, which undermines investor confidence. An increase in external debt, domestic debt, and institutional quality [55] could also reduce FDI inflows.

The application of Driscoll-Kraay standard errors and PCSE produces a robust result. The findings are consistent with previous studies that show that corruption enhances environmental degradation [56,57]. Our result is consistent with other relevant studies that find a significant positive impact of government effectiveness on environmental degradation [58]. This implies that government effectiveness may not reduce environmental degradation unless deliberate action is taken with all seriousness. Also, the current study revealed that political stability significantly positively impacts environmental degradation [59]. Existing studies have found that better regulatory quality may likely reduce environmental degradation [60]. Also, previous studies have suggested that a strict rule of law is likely to reduce the impact of climate change on environmental degradation [61].

The impact of climate change on environmental degradation is negative before and after the implementation of AfCFTA. Also, FDI may contribute negatively to environmental degradation, before the AfCFTA agreement. After the implementation of AfCFTA, FDI may contribute to environmental degradation at a lower rate than before. Also, the result revealed that renewable energy has a significant negative impact on environmental degradation before the AfCFTA agreement. Renewable energy has a significant positive impact on environmental degradation following the implementation of the AfCFTA agreement.

Climate change significantly reduces trade openness, as indicated by the Pool OLS robust, FE estimator, and PCSE. Existing studies have revealed that climate change and trade openness exhibit bidirectional causality [62–64]. Recent studies have found a significant, positive, long-run causal relationship between trade openness and CO2 emissions [65]. Economic growth (GDP per capita growth) has a significant positive effect on trade openness. Relevant studies have indicated that GDP and trade openness are positively related [35,66]. Foreign direct investment (FDI) has a significant positive impact on trade openness. An increase in FDI inflows may accelerate industrialisation, likely boosting trade openness. Our finding is consistent with an existing study [67], which suggested that inflows of FDI are an essential driver of economic development worldwide. Similarly, the current study found that industrialisation is likely to enhance trade openness. The industrialisation has a significant positive impact on trade [68]. Existing studies have investigated the impact of trade openness on industrialisation [43,44,69]. However, there is a paucity of studies examining the impact of industrialisation and economic growth on trade openness in SADC countries.

The impact of climate change on trade openness may be negative before and after the implementation of AfCFTA. While FDI may have contributed positively to trade openness, with about a 12% increase before the AfCFTA agreement. After the implementation of AfCFTA, FDI may increase trade openness by approximately 8%. Also, the result revealed that renewable energy has a significant negative impact on trade openness before the AfCFTA agreement. Renewable energy has a significant positive impact on trade openness following the implementation of the AfCFTA agreement. Considering the institutional factors (qualities), climate change may have a significant negative impact on trade openness. An increase in corruption and all institutional factors may exacerbate the negative effects of climate change on trade openness. The current result is consistent with [70] finding. Similarly, the inflation rate has a significant negative impact on trade openness. Existing studies have found that the inflation rate negatively affects CO2 emissions [71]. It is plausible that an increase in inflation will increase pressure on consumption income and reduce savings [72]. Inflation will reduce purchasing power and affect the quality of life. However, the impact of higher prices on goods and services is likely to harm human behaviour and reduce environmental management and quality.

## 5. Conclusion and policy implications

This study examined the effects of climate change on environmental degradation across 16 selected SADC countries, using data from the World Development Indicators (WDI) and the World Governance Indicators (WDG) for the period 1990 to 2024. Previous studies investigated the impact of climate change on environmental degradation [73,74]. However, there is a paucity of panel analysis in South Africa. All the variables were standardised to avoid spurious analysis results. We used empirical variable selection to create an index of climate change and environmental degradation. The study's pooled OLS may be biased and inconsistent because it assumes no unobserved individual heterogeneity and no omitted time-invariant factors correlated with the regressors [75,76]. Hence, the study employs a fixed-effect estimator that controls for time-invariant heterogeneity and country-specific effects by including robust standard errors or clustering by country identification. Further, the study controls for heteroskedasticity, cross-sectional dependence, and serial correlation by employing Driscoll–Kraay (DK) Standard Errors and PCSE [77,78].

Climate change may hamper trade development in the region. The results showed that the SADC region is climate-sensitive, especially concerning environmental degradation and trade. Both estimation techniques indicate that climate change negatively affects environmental degradation. The study found that while climate change worsens environmental degradation in SADC countries. The results of the current study indicate that continuous increases in ozone depletion could make it more difficult to manage and exacerbate environmental degradation. One consequence of ozone layer depletion is that the weather may be unnecessarily hot, cold, or rainy, leading to irregular seasons unless there is a deliberate, serious policy regulation. Also, from the lens of the AfCFTA regime, both before and after implementation, it shows that climate change contributed to environmental degradation. Logically, it may be expected that FDI should enhance manufacturing and economic growth, thereby mitigating environmental degradation. The study found that FDI negatively impacted environmental degradation. SADC may not attract the expected FDI [79]. The lower inflow of FDI could also be due to institutional factors. Prior to the implementation of AfCFTA, the population may not have affected the environment. However, after the implementation of AfCFTA, the population is likely to have grown, thereby affecting environmental degradation.

Furthermore, the study found that climate change negatively impacted trade openness. As mentioned earlier, industrialisation may have received less FDI in SADC [79]. Notwithstanding, the manufacturing sector could have contributed significantly to the GDP and increased trade. The effects of GDP, industrialisation, and FDI on trade openness do not differ before and after the implementation of the AfCFTA. The results indicate that industrialisation may not influence trade openness after the implementation of AfCFTA. This suggests that there could be more regional trade within SADC. Existing studies have found that common language and common trade influence intra-export in SADC [80]. Also, the elimination of tariffs in 2012 could have contributed to intra-export and sectoral trade [80]. A limitation of the study is that, without an empirical selection of variables to create an index of climate change and environmental degradation, the results may differ. Also, the study did not correct for potential errors arising from using PCA to create the index and to interpolate missing data points. A future study might examine all variables to construct an index of climate change and environmental degradation without empirical justification. The study might also exploit the impact of CO emissions on trade openness in SADC.

The study therefore suggests that governments of SADC countries should adopt measures to mitigate the impact of climate change on broader ecosystems. The region is vulnerable to harsh climatic conditions, which can significantly hamper agricultural production and, in the long run, lead to biodiversity loss and attendant environmental degradation. The SADC countries should consciously reduce carbon emissions and embrace cleaner energy by moving away from relying mainly on fossil fuels. Forest resource preservation should be encouraged to prevent revenue loss from timber exports and help reduce carbon emissions and the incidence of environmental pollution. Sustainable agricultural practices, intra- and extra-trade agreements, and policies that significantly reduce pressure on natural resources. We encourage sustainable consumption, smart waste disposal, and the use of renewable energy to preserve the environment and consequently decelerate the process of climate change, which should be pursued and promoted in SADC.

## Supporting information

S1 Appendix Analysis that included urbanisation. (DOCX)

S2 Appendix Principal component analysis for climate change index variables selection. (DOCX)

S3 Appendix Principal component analysis for environmental degradation index variables selection. (DOCX)
