# Supplementary material for: Climate change and environmental degradation: Evidence from SADC countries
Source: PLoS One. 2026 Apr 6;21(4):e0346018. doi: 10.1371/journal.pone.0346018 (PMC13052876; doi:10.1371/journal.pone.0346018)
Supplement: S1 Appendix — (DOCX) [file pone.0346018.s001.docx]

**Appendix 1**

**Analysis that included urbanisation**

|  | (1) |
| --- | --- |
| Variables | Environmental Degradation |
|  |  |
| Climate change | -0.688*** |
|  | (0.0545) |
| GDP pc growth | 0.665*** |
|  | (0.113) |
| Renewable energy | -1.724*** |
|  | (0.481) |
| lnFDI | 0.629*** |
|  | (0.103) |
| Industralisation | 0.329 |
|  | (0.210) |
| Population | 1.100*** |
|  | (0.129) |
| Urbanisation | 25.00*** |
|  | (7.014) |
| Constant | -82.33*** |
|  | (24.36) |
|  |  |
| Observations | 560 |
| R-squared | 0.663 |

*Robust standard errors in parentheses: *** p<0.01, ** p<0.05, * p<0.1; Authors’ computation, 2026*
