# Supplementary material for: Climate change and environmental degradation: Evidence from SADC countries
Source: PLoS One. 2026 Apr 6;21(4):e0346018. doi: 10.1371/journal.pone.0346018 (PMC13052876; doi:10.1371/journal.pone.0346018)
Supplement: S2 Appendix — (DOCX) [file pone.0346018.s002.docx]

**Appendix 2**

**Principal component analysis for climate change index variables selection**

| Principal components/correlation Number of obs = 560 | | | | | | |  |
| --- | --- | --- | --- | --- | --- | --- | --- |
| Number of comp. = 6 | | | | |  |  |  |
| Trace = 6 | | | |  |  |  |  |
| Rotation: (unrotated = principal) Rho = 1.0000 | | | | | |  |  |
| Component | Eigenvalue | Difference | Proportion | Cumulative |  |  |  |
| Comp1 | 2.51881 | 1.26088 | 0.4198 | 0.4198 |  |  |  |
| Comp2 | 1.25793 | 0.260149 | 0.2097 | 0.6295 |  |  |  |
| Comp3 | 0.997783 | 0.15164 | 0.1663 | 0.7958 |  |  |  |
| Comp4 | 0.846143 | 0.571255 | 0.141 | 0.9368 |  |  |  |
| Comp5 | 0.274888 | 0.170442 | 0.0458 | 0.9826 |  |  |  |
| Comp6 | 0.104446 | . | 0.0174 | 1.000 |  |  |  |
|  |  |  |  |  |  |  |  |
| Principal components (eigenvectors) | | | |  |  |  |  |
| Variable | Comp1 | Comp2 | Comp3 | Comp4 | Comp5 | Comp6 | Unexplained |
| CO_2_ intensity | 0.0357 | -0.038 | 0.9983 | -0.024 | -0.0005 | -0.0036 | 0.000 |
| energy depletion | 0.4452 | 0.5394 | -0.0004 | -0.2798 | -0.3705 | 0.5434 | 0.000 |
| mineral depletion | -0.2311 | 0.7203 | 0.0458 | 0.4056 | 0.5102 | 0.0311 | 0.000 |
| natural resources depletion | 0.5819 | 0.2564 | -0.013 | 0.0363 | -0.0794 | -0.7667 | 0.000 |
| particulate emission damage | 0.5374 | -0.262 | -0.0323 | -0.1793 | 0.7444 | 0.2351 | 0.000 |
| average precipitation in depth | 0.3461 | -0.2333 | 0.000 | 0.8504 | -0.2049 | 0.2462 | 0.000 |
| Principal components/correlation Number of obs = 560 | | | | | | |  |
| Number of comp. = 6 | | | | |  |  |  |
| Trace = 6 | | | |  |  |  |  |
| Rotation: orthogonal varimax (Kaiser off) Rho = 1.0000 | | | | | |  |  |
| Component | Variance | Difference | Proportion | Cumulative |  |  |  |
| Comp1 | 1 | 2.24E-06 | 0.1667 | 0.1667 |  |  |  |
| Comp2 | 1 | 8.02E-07 | 0.1667 | 0.3333 |  |  |  |
| Comp3 | 1 | 2.20E-07 | 0.1667 | 0.5 |  |  |  |
| Comp4 | 1 | 1.17E-06 | 0.1667 | 0.6667 |  |  |  |
| Comp5 | 0.999999 | 2.07E-06 | 0.1667 | 0.8333 |  |  |  |
| Comp6 | 0.999997 | . | 0.1667 | 1.000 |  |  |  |
|  |  |  |  |  |  |  |  |
| Rotated components | | |  |  |  |  |  |
| Variable | Comp1 | Comp2 | Comp3 | Comp4 | Comp5 | Comp6 | Unexplained |
| CO_2_ intensity | 0.000 | 0.000 | 0.000 | 1.000 | 0.000 | 0.000 | 0.000 |
| energy depletion | 0.000 | 1.000 | 0.000 | 0.000 | 0.000 | 0.000 | 0.000 |
| mineral depletion | 0.000 | 0.000 | 0.000 | 0.000 | 1.000 | 0.000 | 0.000 |
| natural resources depletion | 0.000 | 0.000 | 0.000 | 0.000 | 0.000 | 1.000 | 0.000 |
| particulate emission damage | 1.000 | 0.000 | 0.000 | 0.000 | 0.000 | 0.000 | 0.000 |
| average precipitation in depth | 0.000 | 0.000 | 1.000 | 0.000 | 0.000 | 0.000 | 0.000 |
|  |  |  |  |  |  |  |  |
| Component rotation matrix | | |  |  |  |  |  |
|  | Comp1 | Comp2 | Comp3 | Comp4 | Comp5 | Comp6 |  |
| Comp1 | 0.5374 | 0.4452 | 0.3461 | 0.0357 | -0.2311 | 0.5819 |  |
| Comp2 | -0.262 | 0.5394 | -0.2333 | -0.038 | 0.7203 | 0.2564 |  |
| Comp3 | -0.0323 | -0.0004 | 0 | 0.9983 | 0.0458 | -0.013 |  |
| Comp4 | -0.1793 | -0.2798 | 0.8504 | -0.024 | 0.4056 | 0.0363 |  |
| Comp5 | 0.7444 | -0.3705 | -0.2049 | -0.0005 | 0.5102 | -0.0794 |  |
| Comp6 | 0.2351 | 0.5434 | 0.2462 | -0.0036 | 0.0311 | -0.7667 |  |

*Note: Variable selection application: sum (2.51881 + 1.25793) the eigenvalues that are greater than one (1) in the first Table, and divide it by the number of variables (6). This implies that only two (2) should be selected to form the index. Column 1 of the component rotation matrix (last Table) shows that components 1 and 5 have relatively moderate and high degrees. Computed by the Authors’ 2026*
