# Supplementary material for: Climate change and environmental degradation: Evidence from SADC countries
Source: PLoS One. 2026 Apr 6;21(4):e0346018. doi: 10.1371/journal.pone.0346018 (PMC13052876; doi:10.1371/journal.pone.0346018)
Supplement: S3 Appendix — (DOCX) [file pone.0346018.s003.docx]

**Appendix 3**

**Principal component analysis for environmental degradation index variables selection**

| Principal components/correlation Number of obs = 560 | | | | | | |  |
| --- | --- | --- | --- | --- | --- | --- | --- |
| Number of comp. = 6 | | | | |  |  |  |
| Trace = 6 | | | |  |  |  |  |
| Rotation: (unrotated = principal) Rho = 1.0000 | | | | | |  |  |
|  |  |  |  |  |  |  |  |
| Component | Eigenvalue | Difference | Proportion | Cumulative |  |  |  |
| Comp1 | 3.40144 | 1.82696 | 0.5669 | 0.5669 |  |  |  |
| Comp2 | 1.57448 | 0.986648 | 0.2624 | 0.8293 |  |  |  |
| Comp3 | 0.587828 | 0.356113 | 0.098 | 0.9273 |  |  |  |
| Comp4 | 0.231714 | 0.10961 | 0.0386 | 0.9659 |  |  |  |
| Comp5 | 0.122105 | 0.039663 | 0.0204 | 0.9863 |  |  |  |
| Comp6 | 0.082442 | . | 0.0137 | 1.000 |  |  |  |
|  |  |  |  |  |  |  |  |
| Principal components (eigenvectors) | | | |  |  |  |  |
| Variable | Comp1 | Comp2 | Comp3 | Comp4 | Comp5 | Comp6 | Unexplained |
| agricultural land | -0.4223 | -0.2438 | 0.5884 | 0.6441 | -0.034 | -0.0024 | 0.000 |
| arable land | 0.2907 | 0.5905 | -0.3016 | 0.6896 | 0.0003 | -0.0197 | 0.000 |
| forest area | 0.5074 | -0.068 | 0.3323 | 0.0121 | 0.1082 | 0.7847 | 0.000 |
| net forest depletion | 0.4774 | 0.1162 | 0.5133 | -0.0928 | 0.4019 | -0.57 | 0.000 |
| annual freshwater withdrawal | -0.4611 | 0.3534 | -0.0184 | -0.11 | 0.7722 | 0.2317 | 0.000 |
| CO2 emissions | -0.198 | 0.67 | 0.4343 | -0.298 | -0.4788 | 0.0728 | 0.000 |
|  |  |  |  |  |  |  |  |
| Principal components/correlation Number of obs = 560 | | | | | | |  |
| Number of comp. = 6 | | | | |  |  |  |
| Trace = 6 | | | |  |  |  |  |
| Rotation: orthogonal varimax (Kaiser off) Rho = 1.0000 | | | | | |  |  |
|  |  |  |  |  |  |  |  |
| Component | Variance | Difference | Proportion | Cumulative |  |  |  |
| Comp1 | 1.000 | 2.91E-06 | 0.1667 | 0.1667 |  |  |  |
| Comp2 | 1.000 | 1.53E-07 | 0.1667 | 0.3333 |  |  |  |
| Comp3 | 1.000 | -4.42E-08 | 0.1667 | 0.5 |  |  |  |
| Comp4 | 1.000 | 2.83E-06 | 0.1667 | 0.6667 |  |  |  |
| Comp5 | 0.999998 | 3.97E-06 | 0.1667 | 0.8333 |  |  |  |
| Comp6 | 0.999994 | . | 0.1667 | 1.000 |  |  |  |
|  |  |  |  |  |  |  |  |
| Rotated components | | |  |  |  |  |  |
|  |  |  |  |  |  |  |  |
| Variable | Comp1 | Comp2 | Comp3 | Comp4 | Comp5 | Comp6 | Unexplained |
| agricultural land | 0.000 | 0.000 | 1.000 | 0.000 | 0.000 | 0.000 | 0.000 |
| arable land | 0.000 | 0.000 | 0.000 | 1.000 | 0.000 | 0.000 | 0.000 |
| forest area | 1.000 | 0.000 | 0.000 | 0.000 | 0.000 | 0.000 | 0.000 |
| net forest depletion | 0.000 | 0.000 | 0.000 | 0.000 | 0.000 | 1.000 | 0.000 |
| annual freshwater withdrawal | 0.000 | 1.000 | 0.000 | 0.000 | 0.000 | 0.000 | 0.000 |
| CO2 emissions | 0.000 | 0.000 | 0.000 | 0.000 | 1.000 | 0.000 | 0.000 |
|  |  |  |  |  |  |  |  |
| Component rotation matrix | | |  |  |  |  |  |
|  | Comp1 | Comp2 | Comp3 | Comp4 | Comp5 | Comp6 |  |
| Comp1 | 0.5074 | -0.4611 | -0.4223 | 0.2907 | -0.198 | 0.4774 |  |
| Comp2 | -0.068 | 0.3534 | -0.2438 | 0.5905 | 0.67 | 0.1162 |  |
| Comp3 | 0.3323 | -0.0184 | 0.5884 | -0.3016 | 0.4343 | 0.5133 |  |
| Comp4 | 0.0121 | -0.11 | 0.6441 | 0.6896 | -0.298 | -0.0928 |  |
| Comp5 | 0.1082 | 0.7722 | -0.034 | 0.0003 | -0.4788 | 0.4019 |  |
| Comp6 | 0.7847 | 0.2317 | -0.0024 | -0.0197 | 0.0728 | -0.57 |  |

*Note: Variable selection application: sum (3.40144 + 1.57448) the eigenvalues that are greater than one (1) in the first Table, and divide it by the number of variables (6). This implies that only two (2) should be selected to form the index. Column 1 of the component rotation matrix (last Table) shows that components 1 and 6 have relatively moderate and high degrees. Computed by the Authors’ 2026*
